# Treatment of Aphthæ

**Published:** 1871-03

**Authors:** Eustice Smith


### TREATMENT OF APHTHÆ.

BY EUSTICE SMITH, M. D.

Dr. Smith recommends, if *aphthæ* form, that attention be paid to cleanliness. A powder of rhubarb and jalap, with a grain of hydrargyrum cum creta, should be given to evacuate the bowels ; after which the following mixture should be prescribed :

℞—Potass. chloratis, . . . ℈ ij.

Syrupi simpl., . . . ℥ ss.

Aquæ ad . . . ℥ iij. M.

S. ʒ ij. quarta quaque hora.

This must not be diluted. When attacks of acute indigestion come on in infants, with hot skin, furred tongue, thirst, vomiting and diarrhœa, accompanied by griping pain, all food must be stopped, and nothing allowed but cold barley-water. The stomach should be relieved by an emetic of ipecacuanha, after the action of which a purgative of rhubarb and magnesia should be given to clear out irritating matters from the bowels. A mixture of chalk and catechu, with aromatic confection, can then be given, or the following:

| | | |
|---|---|---|
| ℞—Bismuthi nitratis, | . . . | ʒ j. |
| Pulv. cretæ aromat. | . . . | ʒ j. |
| Syrupi, | . . . | ℥ ss. |
| Mucilag. tragacanth, | . . . | ℥ ss. |
| Aquæ ad | . . . | ℥ ij ter die. |

If the diarrhœa continues after the tongue has become clean, half a drop of laudanum can be added to each dose of either of these mixtures, or small doses of sulphuric acid may be given with opium.

| | | |
|---|---|---|
| ℞—Acidi sulphurici aromat., | . . | ʒ ss. |
| Tinct. opii, | . . . . | ♏ vj. |
| Syrupi, | . . . . | ℥ ss. |
| Aquæ carui, ad | . . . . | ℥ iij. ter die. |

When the irritability of the stomach has subsided, milk and lime-water may be given, but with caution, lest the vomiting return.—*Half Yearly Abstract.*

